# Aortic Valve Stenosis and Cardiac Amyloidosis: A Misleading Association

**DOI:** 10.3390/jcm10184234

**Published:** 2021-09-18

**Authors:** Andrea Bonelli, Sara Paris, Matilde Nardi, Michael Y. Henein, Eustachio Agricola, Giovanni Troise, Pompilio Faggiano

**Affiliations:** 1 Cardiology Unit, Spedali Civili and University of Brescia, 25100 Brescia, Italy; bonelliandrea01@gmail.com (A.B.); sara.paris.004@gmail.com (S.P.); matilde.nardi@asst-spedalicivili.it (M.N.); 2 Department of Public Health and Clinical Medicine, Umea University, 90187 Umea, Sweden; michael.henein@umu.se; 3 Cardiovascular Imaging Unit, Cardio-Thoracic-Vascular Department, San Raffaele Hospital, Vita-Salute University, 20132 Milan, Italy; agricola.eustachio@hsr.it; 4 Cardiac Surgery, Cardiothoracic Department, Fondazione Poliambulanza, 25100 Brescia, Italy; giovanni.troise@poliambulanza.it; 5 Cardiology, Cardiothoracic Department, Fondazione Poliambulanza, 25100 Brescia, Italy

**Keywords:** multimodality imaging, amyloidosis, aortic valve stenosis, transthyretin amyloidosis

## Abstract

The association between aortic stenosis (AS) and cardiac amyloidosis (CA) is more frequent than expected. Albeit rare, CA, particularly the transthyretin (ATTR) form, is commonly found in elderly people. ATTR-CA is also the most prevalent form in patients with AS. These conditions share pathophysiological, clinical and imaging findings, making the diagnostic process very challenging. To date, a multiparametric evaluation is suggested in order to detect patients with both AS and CA and choose the best therapeutic option. Given the accuracy of modern non-invasive techniques (i.e., bone scintigraphy), early diagnosis of CA is possible. Flow-charts with the main CA findings which may help clinicians in the diagnostic process have been proposed. The prognostic impact of the combination of AS and CA is not fully known; however, new available specific treatments of ATTR-CA have changed the natural history of the disease and have some impact on the decision-making process for the management of AS. Hence the relevance of detecting these two conditions when simultaneously present. The specific features helping the detection of AS-CA association are discussed in this review, focusing on the shared pathophysiological characteristics and the common clinical and imaging hallmarks.

## 1. Introduction

The coexistence of aortic valve stenosis (AS) and cardiac amyloidosis (CA) is not an uncommon finding in routine clinical practice. AS is the most common valvular heart disease and affects more than 4% of people aged 75 years or older [1]. Similarly, CA has a ≤25% prevalence in octogenarians, according to post-mortem studies [2]. 

Amyloidosis is a rare systemic disorder caused by deposition of amyloid fibrils in different organs including the heart [3]. The two predominant amyloid proteins found in the heart are transthyretin (ATTR) and immunoglobulin light chain (AL) [4] (Table 1), with ATTR amyloidosis the most prevalent form in patients with AS [1]. This combination complicates the diagnostic and therapeutic process. In fact, both conditions share epidemiological, clinical and echocardiographic features, making their management very challenging [5]. Over the last few years, given the sensitivity and specificity of bone scintigraphy, the diagnosis of ATTR-CA significantly increased, without the need for endomyocardial biopsy [6,7]. 

Despite that, the prognostic impact of CA on AS patients is poorly studied, with the few published studies reporting contrasting results. Whether the diagnosis of CA involvement in patients with AS can influence the therapeutic strategies is still a matter of debate, while the newly identified specific pharmacological therapy for ATTR-CA is finding its application in patients with early disease [6].

The aim of this review is to assess the main specifics (epidemiological, pathophysiological, clinical and instrumental) of AS-CA association, which should attract clinicians attention to suspect their coexistence and choose the best therapeutic options. 

## 2. General Features (Epidemiology, Pathophysiology)

Several retrospective or prospective studies have described the presence of ATTR-CA in AS patients (Table 2, Table 3 and Table 4), with a prevalence ranging from 4% to 29% [8]. Such large variability may be explained by heterogeneity of the inclusion criteria and populations investigated. The higher prevalence was found in transcatheter aortic valve replacement (TAVR) cohorts. Interestingly, CA was found in approximately one third of patients undergoing TAVR [5]. 

Transthyretin is synthesized in the liver and has a role as a transporter of thyroxin and retinol-binding proteins. Its main function is the control of behavior, cognition, nerve regeneration and axonal growth [9]. The two common types are wild-type transthyretin (ATTRwt) and variant amyloidogenic (ATTRv) amyloidosis [3]. The former has a prevalence in elderly people, usually with a male preponderance. In this case, the genetic sequence of transthyretin is normal, and the aging process causes protein instability and altered aggregation. On the other hand, ATTRv has a hereditary autosomal dominant transmission, and the pathogenic mutation results in destabilization and misfolding of the ATTR protein. Several mutations have been described [10], and each of them influences the phenotype (i.e., cardiac or neurological predominance) and the severity of the disease. Moreover, the presence of mutation has important consequences on the availability and the use of specific treatment. In contrast, AL amyloidosis is a rare finding in AS patients, mainly because of the poor prognosis of the underlying disease. Indeed, AL amyloidosis is the consequence of a hematological disorder (i.e., multiple myeloma). 

The pathogenetic mechanism of the two types of CA is similar. Over time, the amyloidogenic process results in amyloid fibrils aggregation and precipitation in the extracellular space and consequently causes its expansion. In the heart, this expansion causes increased biventricular wall thickness, myocardial stiffening and restrictive physiology of the left and right ventricles. In addition, amyloid fibrils may exhibit direct toxic effects on myocardial cells, impairing systolic left ventricular function [11]. While this latter mechanism is confirmed for AL amyloidosis, for ATTR it is unproven. In the heart, the progression of amyloid deposition causes severe heart failure and arrhythmias [6].

AS is the consequence of inflammatory process caused by endothelial damage due to mechanical stress and lipid penetration, eventually leading to fibrosis, leaflet thickening, sclerosis and calcification [12]. As a result, the aortic valve orifice size reduces resulting in increased pressure drop (gradient) across the valve [13]. Thus, because of long-standing pressure overload, the left ventricular myocardium thickens, and hypertrophy may mask the recognition of wall infiltration as a potential sign for the presence of an additional infiltrative disease [6]. 

**Figure 1:**
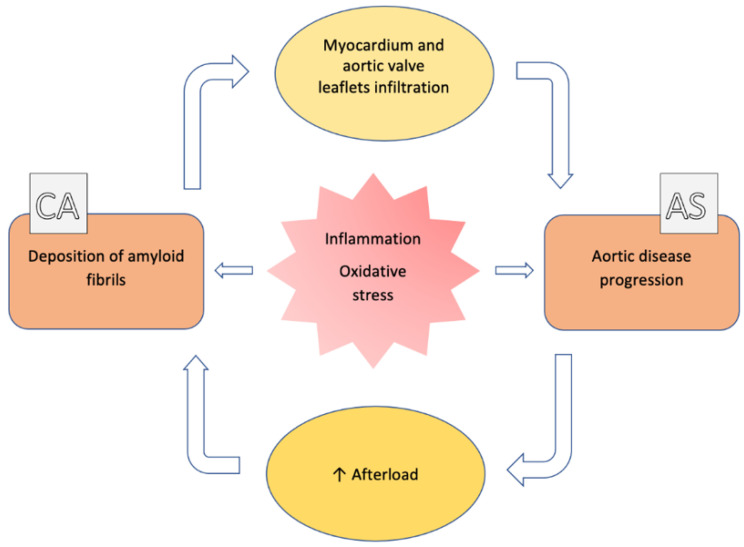
Pathophysiologic correlation between aortic stenosis and cardiac amyloidosis. See the text for details.

Currently, the causative link between AS and ATTR-CA has not been demonstrated. However, there is increasing evidence supporting a central role of oxidative stress, inflammation and extracellular remodeling in the ATTR amyloidogenic process [14]. Some authors postulated that amyloid deposits could be induced or accelerated in AS, because of the pressure overload [15]. On the other side, Kristen et al. reported a high prevalence of amyloid deposits in surgically removed heart valves, mainly in AS (74% of aortic valves) [16], suggesting that amyloid deposits could induce or worsen AS (Figure 1).

**Table 2:** Studies investigating association between aortic stenosis and cardiac amyloidosis. See the text for details.

First Author, Study Year	No. of Patients	Population	ATTR (+), n (%)	AL (+), n (%)	Confirmation of Diagnosis	Management of AS	Follow up Duration	Total No. of Deaths (%)	AS-CA Mortality, n (%)
Treibel, 2016 [17]	146	Severe AS undergoing SAVR	6 (4)	0	EMB, scintigraphy (DPD)	SAVR (146)	2.3 yrs (0.02–4.7)	11 (7.5)	3 (50)
Galat, 2016 [18]	16	Concomitant ATTR and moderate or severe AS	16 (100)	0	EMB, scintigraphy (HMDP/DPD)	SAVR (10), TAVR (2), medical management (4)	33 mos	7 (44)	
Sperry, 2016 [19]	171	Group 1: ATTR (144) Group 2: ATTR + AS (27)	171 (100)	0	EMB, scintigraphy (PYP)	SAVR (11)	6 yrs	58 (34)	10 (37)
Longhi, 2016 [20]	43	Degenerative AS + 1 or more echocardiographic red flags for CA	5 (12)	0	EMB, scintigraphy (DPD)	balloon aortic valvuloplasty	NA	NA	NA
Cavalcante, 2017 [21]	113	Severe and moderate AS scheduled for CMR	9 (8)	0	CMR-LGE	SAVR (42), TAVR (17)	18 mos (11–30)	40 (35)	5 (56)
Castano, 2017 [22]	151	Severe AS undergoing TAVR	24 (16)	0	scintigraphy (PYP)	TAVR	2 yrs	NA	NA
Scully, 2018 [23]	101	Severe AS undergoing TAVR	14 (14)	0	scintigraphy (DPD)	TAVR	NA	NA	NA
Java, 2018 [24]	16	Amyloidosis patients undergoing AVR	5 (31)	6 (38)	EMB or extracardiac biopsy, scintigraphy (PYP)	TAVR (5) SAVR (11)	1.9 yrs (1.2–4.8)	4 (25)	
Peskò, 2019 [25]	55	Retrospective analysis of consecutive amyloidosis patients	9 (20)	44 (80)	CMR-LGE, scintigraphy (PYP)	NA	NA	9 mos mortality:23 (42)	NA
Nitsche, 2020 [26]	191	Severe AS undergoing TAVR	15 (8)	1 (0.5)	CMR, scintigraphy (DPD), EMB	TAVR	15.3 ± 7.9 mos	33 (17)	3 (19)
Scully, 2020 [27]	200	Severe symptomatic AS referred for TAVR	26 (13)	0	scintigraphy (DPD)	TAVR (149), SAVR (2), medical management (49)	19 (10–27) mos	42 (21)	6 (23)
Nitsche, 2021 [6]	407	Severe AS undergoing TAVR	47 (11)	1 (0.2)	scintigraphy (DPD), EMB or extracardiac biopsy	TAVR (333), SAVR (10), medical management (65)	1.7 yrs	97 (24)	1 year mortality: 15 (31)
Rosenblum, 2021 [28]	204	Severe AS undergoing TAVR	27 (13)	0	scintigraphy (PYP)	TAVR	2.04 yrs	63 (31)	9 (33)
Faggiano, 2021 (unpublished data)	50	Patients with clinically relevant aortic stenosis	9 (18)	0	scintigraphy (DPD)	TAVR, SAVR, medical therapy	NA	NA	NA

Legend: AVR: aortic valve replacement; CMR: cardiac magnetic resonance, DPD: ^99m^Tc-3,3-diphosphono-1,2-propanodicarboxylic-acid; EMB: endomyocardial biopsy; HMDP: ^99m^Tc-hydroxymethylene diphosphonate; LGE: late gadolinium enhancement, NA: not available; SAVR: surgical aortic valve replacement; PYP: ^99m^Tc-pyrophosphate; TAVR: transcatheter aortic valve replacement.

**Table 3:** Clinical characteristics and laboratory findings of AS-CA patients. See the text for details.

First Author, Study Year	Age, Years	Male (%)	NYHA I/II/III/IV, (%)	AF (%)	Carpal Tunnel Syndrome, n (%)	Nt-proBNP (ng/dL)
Treibel, 2016 [17]	77	67	NA	NA	1 (17)	259
Galat, 2016 [18]	79 ± 6	81	III–IV (60)	56	5 (31)	438 (243–473)
Sperry, 2016 [19]	79.4 ± 6.6	71	III–IV (66.6)	58.3	NA	NA
Longhi, 2016 [20]	84 (79–90)	80	III–IV (100)	NA	3 (60)	NA
Cavalcante, 2017 [21]	88 ± 6	89	III–IV (78)	67	NA	NA
Castano, 2017 [22]	86.3 ± 5.7	92	0/25/75/0	41.7	4 (16)	3220 (1092 ± 19,007)
Scully, 2018 [23]	88 ± 6	50	NA	NA	NA	NA
Java, 2018 [24]	76 (71–82)	69	6/50/31/13	13	NA	NA
Peskò, 2019 [25]	69 (68–82)	60	III–IV (100)	20	NA	NA
Nitsche, 2020 [26]	84 (81–89)	63	III–IV (62.5)	56.3	NA	3634 (1241–6323)
Scully, 2020 [27]	88 ± 5	62	NA	42	NA	370 (129–563)
Nitsche, 2021 [6]	87 (84–92)	65	NA	50	6 (19)	486 (141–750)
Rosenblum, 2021 [28]	86 ± 5	96	III–IV (100)	37	6 (22)	NA
Faggiano, 2021 (unpublished data)	85.6 ± 4.3	84	II (44)–III/IV (55)	77	1 (11)	NA

Legend: AF: atrial fibrillation; Nt-proBNP: N-terminal pro-brain natriuretic peptide; NYHA: New York heart association; NA: not available.

**Table 4:** Imaging parameters of aortic stenosis-cardiac amyloidosis patients. See the text for details.

First Author, Study Year	LVEF (%)	IVST, mm	LF-LG AS, %	AVA, cm^2^	AVAi, cm^2^/m^2^	Mean AV Gradient (mmHg)	LV SV Index mL/m^2^	GLS (%)	CMR LGE (+), n (%)
Treibel, 2016 [17]	67	16.7	NA	NA	0.4	NA	NA	−12.6	2 (30%)
Galat, 2016 [18]	50 ± 13	18 ± 4	86	0.8 ± 0.25	NA	33 ± 23	27 ± 7	−7 ± 0.7	12 (100%)
Sperry, 2016 [19]	50 ± 13.9	18.6 ± 4.4	40.7	0.89 ± 0.29	0.45	21.8 ± 13	NA	NA	NA
Longhi, 2016 [20]	↓ in 40%	18 (16–21)	80	NA	< 0.6	NA	NA	NA	NA
Cavalcante, 2017 [21]	43 ± 17	18 ± 5	78	NA	0.4 ± 0.2	30 ± 14	33 ± 10	NA	9 (100%)
Castano, 2017 [22]	47.6 ± 17.6	13 ± 0.3	37.5	NA	0.8 ± 0.16	35.2 ± 13.9	29.9 ± 10.5	−12.4 + 5.2	NA
Scully, 2018 [23]	NA	NA	NA	NA	NA	37 ± 12	32 ± 7	NA	NA
Java, 2018 [24]	60 (59–65)	13 (11–14)	NA	NA	0.51	46 (36–51)	NA	NA	NA
Peskò, 2019 [25]	59 (51–60)	17 (13–20)	60	NA	NA	NA	30	NA	3 (60)
Nitsche, 2020 [26]	62 (44–70)	15.5 (13.3–19.8)	56.3	0.6	NA	35 (26–48.5)	27.4 (22.3–33.7)	−13.8 (16.6–10.2)	4 (25%)
Scully, 2020 [27]	54 ± 14	14 ± 3	31	0.74 ± 0.23	NA	37 ± 14	34 ± 10	−15 ± 6	NA
Nitsche, 2021 [6]	51 (42–64)	16 (14–19)	56	0.7	NA	36 (25–48)	35.8 (27.4–44)	−13.7 (17.3–10.2)	NA
Rosenblum, 2021 [28]	48 ± 17	14 ± 4	37	0.8 ± 0.15	NA	35 ± 13	31 ± 11	NA	NA
Faggiano, 2021 (unpublished data)	42.4 ± 9.9	18.3 ± 4.1	NA	0.64 ± 0.12	NA	33.4 ± 22.5	NA	NA	NA

Legend: AV: aortic valve; CMR: cardiac magnetic resonance; GLS: global longitudinal strain; IVST: interventricular septal thickness; LF-LG: low flow - low gradient; LGE: late gadolinium enhancement; LVEF: left ventricular ejection fraction, LV SV: left ventricular stroke volume; NA: not available.

## 3. Clinical and Imaging Assessment

Diagnosis of ATTR-CA in AS patients is challenging, as clinical and imaging features can overlap. Moreover, comorbidities such as coronary artery disease and hypertension are frequently present in the older population, making the diagnostic process, based on any risk score, even more difficult [5]. 

Researchers have assessed the clinical and imaging features of patients with concomitant AS and ATTR-CA (Table 3 and Table 4). Firstly, ATTR-CA is more often found in male patients, and its prevalence significantly grows with increasing age [6,22]. These patients often have a clinical history of carpal tunnel syndrome [29,30], lumbar spinal stenosis [31], biceps tendon rupture, deafness [32], premature pacemaker implantation and disproportionate heart failure symptoms despite non-severe AS. One typical characteristic is the presence of predominant signs of right ventricular failure. On the other hand, macroglossia, a well-known manifestation of AL amyloidosis, is less frequent in ATTR-CA [8]. 

Another clue is the so-called “natural cure” of hypertension with the need for down-titration or discontinuation of antihypertensive medications. This reflects the presence of an autonomic dysfunction. Moreover, the presence of unexplained peripheral or autonomic neuropathy suggests the possibility of hereditary ATTR amyloidosis but can occur in AL and occasionally in ATTRwt amyloidosis [33]. 

Some laboratory parameters are suggestive of CA including disproportionate N-terminal pro-brain natriuretic peptide (NT-proBNP) elevation with AS severity and in the absence of chronic renal failure or elevated troponin levels without significant coronary artery disease or renal failure [8]. Unlike AL amyloidosis, there are no specific circulating biomarkers of ATTR-CA, although the endogenous transthyretin ligand retinol binding protein-4 (RBP4) has shown promising results [34].

Patients with ATTR-CA rarely have normal electrocardiography (ECG). Two criteria are suggestive of CA: pseudo-infarction pattern (mainly in anterior leads) and low-voltage QRS complex. The former is common and has been observed in approximately 60% of patients with ATTR-CA [35]. Conversely, the pathognomonic low voltage pattern has been demonstrated only in 25–40% of patients [36,37]. Indeed, from 7 to 10% of patients with ATTR-CA may show LV hypertrophy on ECG [38]. 

As the deposition of amyloid fibrils progresses over time, direct involvement of the sinoatrial node, atrioventricular node and bundle branches can manifest in the form of various degrees of conduction abnormalities and arrhythmias, such as atrial fibrillation. These abnormalities tend to become more frequent with increasing disease severity [39]. Atrial fibrillation is the most common arrhythmia, affecting 41 to 67% of patients with concomitant AS and ATTR-CA. 

Transthoracic echocardiography is essential in the diagnostic process of both AS and CA. The assessment of aortic valve disease should follow current guidelines. The critical point is that both pathologies share some characteristics, such as LV wall thickening, impaired diastolic function (as reflected on filling pattern), and/or LV systolic dysfunction [8]. An enlarged left atrium will also support the presence of left ventricular stiffness and will explain the mechaqnism of atrial arrhythmias. 

Some Doppler echocardiographic features are mainly observed in CA patients: concomitant left and right ventricular wall thickening, bi-atrial dilation, mild pericardial effusion, thickening of atrioventricular valves and atrial septum, and restrictive left ventricular filling pattern [40]. About 79% of ATTR-CA patients have an asymmetric pattern of increased left ventricular wall thickness, as shown in echocardiographic and cardiac magnetic resonance studies, instead of the typical concentric pattern [35,41]. All these features may be absent at an early stage of the disease, and the increased myocardial echogenicity, also called ‘granular sparkling’, often becomes apparent at the late stage of the disease [35]. Recently, two multiparametric scores have been proposed with good sensitivity and specificity to either diagnose or exclude CA in two clinical scenarios: among patients with proven systemic AL amyloidosis or in patients with a hypertrophic cardiac phenotype [42]. The AL score includes relative wall thickness (RWT), tricuspid annular plane systolic excursion (TAPSE), E wave e’ wave ratio (E/e’) and longitudinal strain with an AUC (area under the curve) of 0.909 while the increased wall thickness (IWT) score also included septal longitudinal systolic apex-to-base ratio with an AUC of 0.870. When compared to isolated AS, patients with concomitant AS and ATTR-CA have greater LV wall thickness, higher LV mass index [21] and a worse degree of diastolic dysfunction [22]. Typically, ATTR-CA patients have low-flow low-gradient AS [18,20,21,22].

**Figure 2:**
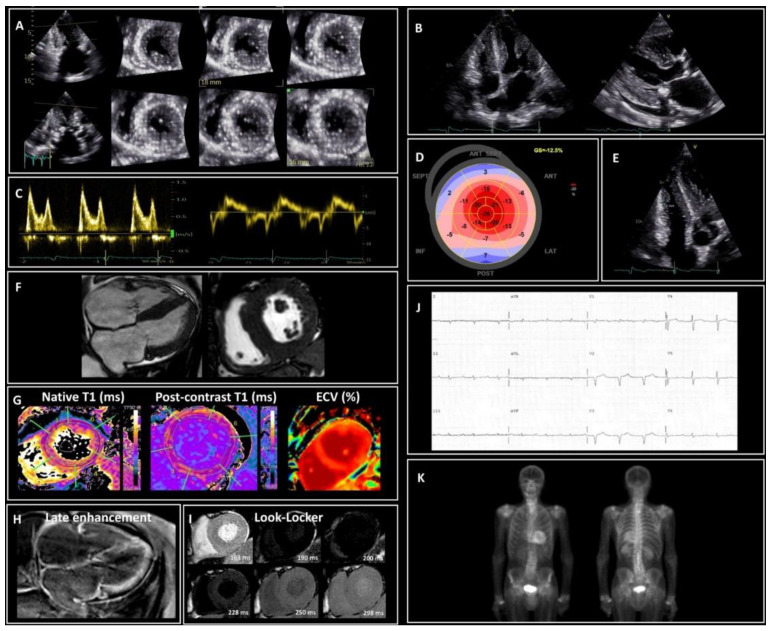
Red flags of cardiac amyloidosis (CA). (**A–E**) Typical echocardiographic features of CA. (**A**) Left ventricle (LV) short-axis slices obtained from a 3D dataset showing severe LV hypertrophy and myocardial granular sparkling. (**B**) 4-Chamber view and parasternal long-axis showing an increase of left and right ventricular walls thickness, left atrial dilatation, atrial septal thickening and pericardial effusion. (**C**) Pulsed wave Doppler of the mitral valve inflow reveals restrictive filling pattern (grade III diastolic dysfunction) with marked reduced lateral mitral annular diastolic velocity (e′ 4.8 cm/sec at the tissue Doppler analysis); tissue Doppler signals from the lateral mitral annulus shows longitudinal systolic dysfunction with mitral Sʹ ≤ 6 cm/s. (**D**) Speckle tracking imaging showing a depressed LV global longitudinal strain (−12%) with apical sparing and an apex/basal longitudinal strain ratio > 2. (**E**) Focused right ventricle (RV) view showing RV wall thickening (≥5 mm). (**F**–**I**) Typical cardiac magnetic resonance (CMR) features of CA. (**F**) Steady-state free precession sequences (SSFP) showing diffuse and asymmetric hypertrophy of the LV and RV. (**G**) T1-mapping reveals prolongation of the native relaxation time and of the extracellular volume (ECV). (**H**) Typical late gadolimium enhancement (LGE) pattern: LGE is extensive and circumferential, starts from the subendocardium and predominates at the basal segments with a base-to-apex gradient in a non-ischemic pattern; sub-optimal nulling of myocardium is present, and the blood pool has a signal darker than the myocardium; LGE is also evident in the RV wall, atria walls and atrial septum. (**I**) Frames from Look–Locker inversion recovery sequences (T1 scout) showing altered gadolinium kinetics in ATTR-CA: evidence of reverse order of sequences with the myocardium passing through the null-point before the blood pool. (**J**) Typical ECG findings of CA: discordance between low-voltage and LV wall thickness; discordance between the voltages in peripheral and precordial leads; pseudo-infarction pattern (Q waves) without history of myocardial infarction; right axis deviation; abnormal P wave duration and morphology reflecting slow atrial conduction. (**K**) ^99m^Tc-hydroxymethylene diphosphonate scintigraphy showing strong cardiac uptake (Perugini Grade 2).

The analysis of myocardial deformation by tissue Doppler and speckle-tracking echocardiography has a crucial role in the detection of CA [43]. Reduced LV longitudinal deformation is an early marker of cardiac amyloid deposition [44,45] (Figure 2). Significantly reduced LV longitudinal deformation and global longitudinal strain has been recently reported in patients with concomitant ATTR-CA and AS [17,18] compared to lone AS. The typical pattern of ‘apical sparing’ refers to the abnormal ratios of apical to basal strain or apical to basal plus midventricular strain, which is specific in CA. The pattern reflects the more normal strain values of left ventricular apical region compared to progressively worse values at the mid and basal regions. Apical sparing has shown good diagnostic accuracy for differentiating amyloid heart disease from other etiologies [33]. Interestingly, in the study by Castano et al. [22], the apical sparing pattern was not depicted in patients with ATTR-CA and AS. This is due to elevated wall stress and increased afterload induced by AS that finally masks the reduced apical deposition of amyloid in comparison to other segments. Thus, the discriminatory power of speckle-tracking in patients with dual pathology should be elucidated, because a classical apical-sparing pattern may be hidden by the presence of AS. In contrast, the apical sparing may also be observed in patients with AS and no CA [8], with evidence suggesting that relative apical sparing becomes manifest only after TAVR [22].

Cardiac imaging using magnetic resonance (CMR) has an important diagnostic role in CA. It carries additional diagnostic value because of the better myocardial tissue characterization with late gadolinium enhancement (LGE), native T1 mapping, and extracellular volume (ECV) sequences [33]. However, clinicians have to consider that 15% of CMR examinations may be normal in patients with CA [46]. LGE on CMR reflects the interstitial expansion secondary to amyloid deposition. The typical finding is circumferential LGE within the entire LV sub-endocardium with a various degrees of myocardial extension and a base-to-apex gradient [47]. The detection of a global transmural LGE is associated with a greater interstitial amyloid deposition on myocardial histology [48] (Figure 2). Moreover, elevated native myocardial T1 and extracellular volume in ATTR-CA carries a high diagnostic accuracy, in detecting CA when conventional clinical testing and LGE are normal [49]. Native T1 and ECV values have shown progressive correlation with increasing cardiac amyloid burden [50]. Patients with concomitant ATTR-CA and AS have been shown to exhibit higher native T1 and ECV values, compared to patients with isolated AS [6,21]. 

Once ATTR-CA is suspected, the next step is to confirm the diagnosis. The 2019 expert consensus for the suspicion and diagnosis of ATTR-CA allows to avoid endomyocardial or extra-cardiac biopsy (fat pad biopsy) if bone scintigraphy is available [51]. Furthermore, endomyocardial biopsy may not be appropriate in elderly and fragile patients. However, in some cases, biopsy of the involved organ must be performed, if the clinical suspicion is high. 

Performing bone scintigraphy with 99m technetium-labeled bisphosphonates and excluding AL by search of monoclonal light chain in blood and urine is now sufficient to confirm the suspicion of ATTR-CA [52]. In particular, grade 2 or 3 uptake on scintigraphy, using the Perugini score [53] and the absence of a monoclonal protein have specificity and a positive predictive value of 100% for ATTR-CA [52] (Figure 2). Then, genotyping is required to distinguish ATTRwt from ATTRv [18]. Clinicians should not forget that 30–50% of AL-CA patients also display cardiac uptake on scintigraphy, typically a grade 1 uptake [54]. As already stated, patients with AS mostly have ATTR-CA; however, AL-CA should be always excluded with serum/urine light chain protein analyses, because of its poor prognosis in the absence of chemotherapy [8].

**Figure 3:**
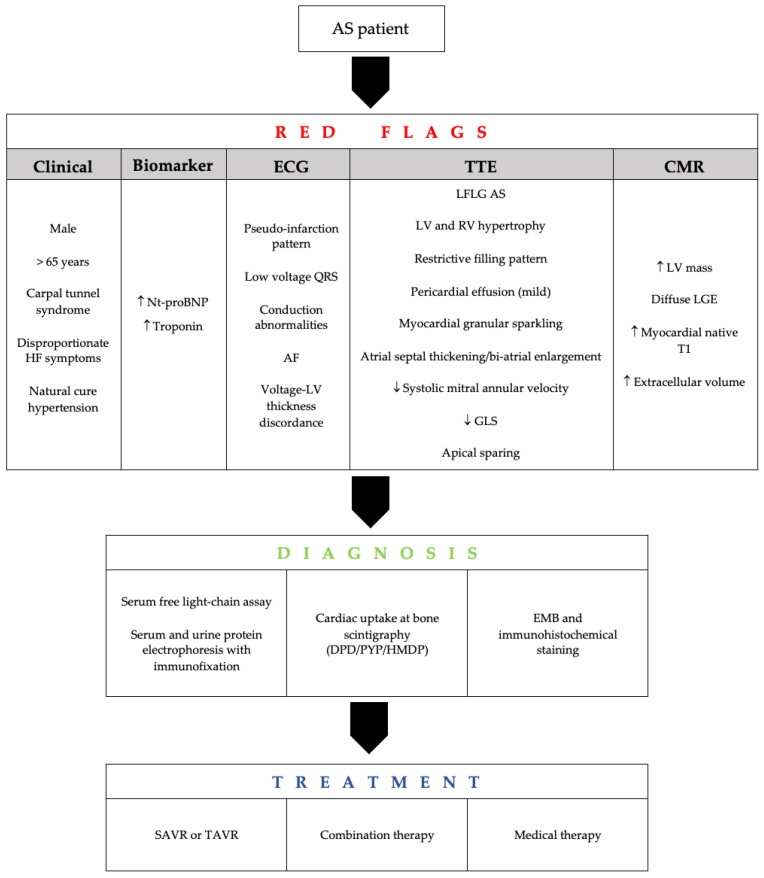
Diagnostic flow-chart and red flags to recognize cardiac amyloidosis involvement in AS patients. See the text for details. Legend—AF: atrial fibrillation; AS: aortic stenosis; DPD: 99mTc-3,3-diphosphono-1,2-propanodicarboxylic-acid; EMB: endomyocardial biopsy; GLS: global longitudinal strain; HF—heart failure; HMDP: 99mTc-hydroxymethylene diphosphonate; LFLG: low-flow low-gradient; LGE: late gadolinium enhancement; LV: left ventricle; Nt-proBNP: N-terminal pro-brain natriuretic peptide; PYP:99mTc-pyrophosphate, SAVR: surgical aortic valve replacement; TAVR: transcatheter aortic valve replacement.

Although endomyocardial biopsy and confirmation using mass spectrometry remain the gold standard for diagnosing ATTR-CA, these procedures often delay diagnosis and may not be appropriate in frail elderly adults, including those referring for TAVR. In the absence of specific CA features on imaging, a positive extracardiac biopsy is not sufficient to confirm the diagnosis. In contrast, a negative extracardiac biopsy may not exclude CA in patients with typical CA features on imaging [55]. A diagnostic flow-chart is proposed in Figure 3. 

## 4. Screening and Predictors

To date, there is no recommendation on whether patients with AS should be systematically screened for CA [8]. However, patients with AS requiring TAVR should be screened if there is a suspicion of concomitant ATTR-CA [5]. Recent studies have shown that patients with concomitant AS and CA are older than those with lone AS [6,22,26,27]. Age is also a significant predictor of ATTR-CA among patients referred for TAVR, even after adjustment for other variables [26,27] A history of carpal tunnel syndrome, especially if bilateral, may independently indicate the presence of amyloid deposits of ATTR [6]. NT-proBNP and high sensitivity cardiac troponin (hs-cTn) before TAVR should always be assessed, as they have been shown to be double in patients with concomitant CA and AS compared to those with lone AS, irrespective of renal function. Cardiac troponin has also been found to have a potential predictive value in this setting, but ranges were too wide to choose a cut-off [27].

ECG is another important marker of cardiac structure and function in CA and AS. Patients with concomitant ATTR-CA and AS usually exhibit more pronounced ECG abnormalities than those with solely AS. They tend to have broader QRS complex and higher prevalence of right bundle branch block B [6,22]. Both these features showed a good predictive power of AS-CA at multivariate analysis [26,27]. In this context, the discordance between QRS voltage and LV hypertrophy on imaging is considered a valid element of suspicion and should help differentiating CA from hypertensive or hypertrophic cardiomyopathy [33]. Using Sokolow–Lion index to suspect cardiac involvement in patients with AS may also be helpful, as a predictor of the association between CA and AS [26,27]. This condition is also represented by another parameter, the voltage/mass ratio (VMR). The VMR combines LV mass index and signs of hypertrophy on ECG [56]. VMR values are usually lower in CA-AS patients, and this parameter has been found to efficiently discriminate between lone AS and CA-AS [6,26,27]. However, attention should be paid in case of bundle branch block or pacemaker-induced rhythm because of poor reliability on voltage quantification. 

Once raised a suspicion, echocardiography with myocardial deformation analysis may add discriminatory power for the presence of CA, as initial imaging technique. Interestingly, Nitsche and colleagues showed that apical sparing was a powerful marker for diagnosing AS-CA [26]. AS-CA patients appeared to have significantly lower LV ejection fraction (LVEF), lower stroke volume index and lower trans-aortic gradient [18,20,21,22]. All these echocardiographic parameters together with high grade diastolic dysfunction, concentric hypertrophy and increased left atrial volume showed predictive power on univariate analysis [22,27]. However, only the systolic mitral annular velocity (S’) was the best predictor of ATTR-CA in a multivariable logistic regression analysis, with an AUC of 0.95, *p* < 0.0001, compared to the rest of the echocardiographic variables. Indeed, a cut-off value of S’ < 6 cm/s had 100% sensitivity (with a 57% specificity) in predicting a positive 99mTc-PYP (99mTc-labeled pyrophosphate) amyloid scan [22]. Thus, S’ may be used by clinicians as a valid tool for screening. Stroke volume index (SVi) was also proposed as an independent predictor of CA in patients with AS. As for low mitral annular systolic velocity, SVi values have been able to detect CA-AS with an AUC of 0.77, suggesting an additional useful tool. This finding reflects typical low flow aortic pattern for CA-AS patients [26]. The high prevalence of low-flow state is secondary to several factors: LV concentric remodeling, impairment of diastolic filling, left atrial remodeling and dysfunction and RV remodeling and dysfunction. However, 50% of patients with CA and low-flow, low-gradient AS have preserved LVEF. In this setting, stress echocardiography may be used to confirm AS severity [57]. Sometimes, in patients with CA, it may provide inconclusive results, when it fails to significantly increase LV outflow velocities [8]. In this case, the quantitation of aortic valve calcium burden using non-contrast CT may be a valid option to confirm AS severity [58].

Cardiac magnetic resonance (CMR) has demonstrated promising value for disease detection, following disease progression or monitoring response to therapy in patients with CA. However, Nitsche et al. confirmed the formerly reported low sensitivity of distinctive LGE patterns. In this study, CMR was diagnostic in only 25% of CA-AS patients [26]. Given the high cost, the availability of CMR and available findings, CMR does not seem a suitable screening tool in patients referring for TAVR.

In a recent study, Nitsche and colleagues created and validated in two cohorts a scoring system for discrimination of lone AS versus AS-CA. The remodeling, age, injury, system and electrical (RAISE) score includes 5 domains: remodeling (LV hypertrophy and/or diastolic dysfunction), age, injury (hs-cTn), systemic (carpal tunnel syndrome) and electrical (right bundle branch block or low voltages). Scores ≥2 and ≥3 points had high sensitivity (93.6% and 72.3%), with adequate specificity (52.1% and 83.6%) for the presence of AS-CA, suggesting a potential additional valid screening tool [6].

## 5. Prognosis and Management

Combination of AS with CA is prognostically important. Currently, only few studies have investigated the outcome of AS-CA patients and have shown worse prognosis. Treibel and colleagues have followed 146 patients with severe AS who underwent surgical AVR for a median of 2.3 years. Of all variables assessed, the presence of ATTR-CA had the highest hazard ratio for all-cause mortality [17]. Similarly, in another group of older AS patients, CA was associated with significantly increased 1-year all-cause mortality, independently of aortic valve disease treatment. Even after adjustment for other variables including aortic valve replacement (i.e., Surgical Thoracic Society Predicted Risk of Mortality, LV ejection fraction with CMR and NYHA functional class), the presence of CA in elderly AS patients was associated with increased all-cause mortality [21]. Nevertheless, Sperry et al. found no significant difference in the 2-year mortality rate between ATTR-CA patients with and without AS [19]. Given these contrasting results, further studies are necessary to evaluate how much ATTR-CA affects outcomes, in order to choose the best therapeutic approach, especially in the TAVR era. To date, there are no recommended guidelines and no expert consensus that point out the best management of CA in patients with AS [24]. 

Most studies assessing patients following surgical aortic valve replacement showed a high risk of mortality and modest improvement in functional class [17,18,19,21,59,60]. Thus, it seems reasonable to prefer TAVR in AS-CA patients, rather than open heart surgery as some small studies suggested better outcomes with this approach [18,26]. In fact, surgical replacement has been described to have higher risk for several peri-procedural complications, including fatal arrhythmias, progressive heart failure and myocardial infarction with mechanical complications [17,60,61]. Despite that, TAVR is not risk free, with reports showing LV rupture and complete atrioventricular block leading to death during or after the TAVR in patients with CA [59,62]. 

In view of the above, individual cases should be discussed by a multidisciplinary team in order to choose the best treatment options (i.e., surgical AVR, TAVR or medical treatment) [8]. Depressed LVEF (<50%), severely reduced global longitudinal strain (GLS < −10%), restrictive filling pattern, moderate-to-severe low-flow state (stroke volume index < 30 mL/m^2^) and low-gradient AS [17,18] have been shown to be associated with poor clinical outcome. The availability of specific treatment for ATTR amyloidosis has also changed the natural history of the disease to the point that medical therapy is considered a valid treatment option, also in patients with AS. Several disease-modifying therapies for ATTR amyloidosis are now available, based on early diagnosis [5]. Selection of best therapy is complex, but each drug has particular indications that may guide the choice. The 2021 guidelines for the diagnosis and treatment of acute and chronic heart failure recommend the use of tafamidis for both NYHA class I or II ATTRwt and ATTRv patients in order to reduce symptoms, cardiovascular hospitalizations and mortality (class of recommendation I, level of evidence B) [63]. The nonsteroidal anti-inflammatory agent diflunisal may be an option, but its use is still off-label. On the other hand, the ATTR silencer patisiran and inotersen have been approved only in presence of proven polyneuropathy [32]. In fact, patisiran may be considered for those with ATTRv and polyneuropathy [63]. Thus, specific treatment should be commenced as soon as ATTR-CA is confirmed and administered in parallel to other HF therapies, irrespective of the need for a valve replacement procedure [8]. 

## 6. Conclusions

The combination of AS and CA, especially ATTR, is an important clinical problem. Its already high prevalence is destined to grow because of the aging population. However, current literature shows that CA is often underdiagnosed in old adults, resulting in underestimation of the AS-CA combination. Such clinical issue has its impact on screening patients with AS for the presence of CA and the choice of best treatment strategy. We highlighted some clinical and imaging features which should help raise the suspicion of cardiac amyloid involvement, followed by diagnosis confirmation with bone scintigraphy. They should be followed by individual case multidisciplinary assessment to evaluate the best suitable approach. Based on the available uncertainty of the clinical outcome of combined AS and CA, prospective multicenter studies in large cohorts are necessary to suggest an optimum road map for managing such patients. 

## Figures and Tables

**Table 1:** Main amyloid types with possible cardiac involvement.

Fibril Protein	Precursor Protein	Target Organs
ATTR	Transthyretin (wild-type or variant)	Wild type: heart, carpal tunnel syndrome (bilateral), ligaments, lumbar spinal stenosis Variant (variable): heart, PNS, ANS, ligaments, lumbar spinal stenosis, leptomeningeal, eye, gastrointestinal tract
AL	Monoclonal immunoglobulin light chain	Heart, PNS (no CNS), ANS, liver, lung, gastrointestinal tract, soft tissues (tongue), kidney, myopathy

Legend: ANS: autonomic nervous system; CNS: central nervous system; PNS: peripheral nervous system.
